# Development of the Bristol Rabbit Pain Scale (BRPS): A multidimensional composite pain scale specific to rabbits (*Oryctolagus cuniculus*)

**DOI:** 10.1371/journal.pone.0252417

**Published:** 2021-06-11

**Authors:** Livia Benato, Joanna Murrell, Toby G. Knowles, Nicola J. Rooney

**Affiliations:** 1 Animal Welfare and Behaviour, School of Veterinary Sciences, University of Bristol, Langford, United Kingdom; 2 Highcroft Veterinary Referrals, Whitchurch, Bristol, United Kingdom; University of Lincoln, UNITED KINGDOM

## Abstract

A species-specific composite pain scale is a prerequisite for adequate pain assessment. The aim of this study was to develop a multidimensional pain scale specific to rabbits (*Oryctolagus cuniculus*) called the Bristol Rabbit Pain Scale (BRPS). The scale was developed over five phases using a unique combination of methods: focus groups and behavioural observation. The first two phases aimed at identifying descriptors to describe a rabbit in pain, and then reducing their number, both using focus groups. A total of 72 pain descriptors were grouped under six categories (Demeanour, Posture, Facial expression, Attention to the painful area, Audible and Other) and ‘No pain’ descriptors were added. The third phase aimed to confirm, through video observation of rabbits, the categories and descriptors previously described, to reject those terms that were ambiguous, and identify any new descriptors that had not been included in the previous list of descriptors. This led to the rejection of the categories Audible and Attention to the painful area and of 34 descriptors. Seven new descriptors were identified. The last two phases constructed the final format of the BRPS by refining the categories, ranking the descriptors on an ordinal scale and testing the internal reliability of the scale using Cronbach’s alpha test. This led to a composite pain scale of six categories (Demeanour, Posture, Locomotion, Ears, Eyes and Grooming) with four intensities of pain (0, 1, 2, and 3), a total score of 0–18, and a high Cronbach’s alpha coefficient (alpha = 0.843). This BRPS fills an important gap in the field of rabbit medicine and has the potential to improve the assessment and management of pain in rabbits providing veterinary professionals with a novel multidimensional pain assessment tool. Further studies will investigate the clinical utility, validity and reliability of the BRPS.

## Introduction

In veterinary clinics in the UK, many surgical procedures such as dental treatment, lump and abscess removal and elective surgeries such as ovariohysterectomy and orchiectomy are regularly performed on rabbits (*Oryctolagus cuniculus*) [1, 2]. More than 11,000 experimental procedures are also carried out on this species in laboratory settings (Home Office, Annual statistics of scientific procedures on living animals Great Britain 2018). Adequate management of perioperative pain is therefore essential. The ability to ameliorate pain relies on the capacity to detect and quantify it adequately [3].

The Rabbit Grimace Scale (RbtGS) is a pain scale specifically designed for rabbits [4]. The RbtGS relies on five facial indicators (Orbital tightening, Cheek flattening, Nostril shape, Whisker shape and position, and Ear shape and position) each on a 0–2 scale; where 0 is no pain and 2 is extreme pain. The RbtGS is easy to use, although some of the facial indicators such as whisker position may be difficult to assess [5]. Moreover, it has been developed based on a breed of straight-eared rabbits, common in laboratories. This can be a limiting factor when used in a clinical setting as lop-eared rabbits currently comprise the majority of the cases treated in UK veterinary practices [6].

Grimace pain scales are very popular pain assessment tools, because they are reliable and quick to use in a busy clinical environment [7]. However, the experience of pain is multi-dimensional and composite pain scales are considered the gold standard when assessing pain [8]. More recently, a new composite pain scale specific for rabbits (CANCRS) has been designed, merging the Rabbit Grimace Scale (RbtGS) with a clinical pain scale (CPS) comprising physiologic parameters (pupil dilation, respiratory rate, respiratory pattern, heart rate) and behavioural responses (response to palpation, mental status and vocalization) [9]. The scale rates four levels of pain intensity (No pain, Discomfort, Moderate pain and Severe pain). The overall scale (CANCRS), and the two component elements: (RbtGS and CPS) were each tested for reliability and validity. The results showed that both elements are reliable assessment tools in a clinical environment while the CPS did not differentiate between the different levels of pain. Physiological parameters can present limitations when assessing pain, as they can be altered by concurrent problems such as stress and infection [10–12].

In 1985, Morton and Griffiths [13] developed a multidimensional pain scale for use in a laboratory context. This pain scale took in consideration changes due to pain in a group of laboratory animals: rabbits, monkeys, dogs, cats, guinea pigs, and rats [13]. The scale was based on the clinical signs seen most commonly by the animal carers such as veterinary surgeons, veterinary nurses or research workers and described five categories (body weight, appearance, clinical signs, unprovoked behaviour, and response to an appropriate stimulus). A score of 0 to 3; where 0 is normal and 3 is severe; was then assigned to each category. This composite pain scale has not been tested for reliability and validity, and it is not specific to rabbits, possibly leading to a misinterpretation of the clinical signs of pain in this species.

The method used by Morton and Griffiths (1985) based on focus group discussion was also used to develop species-specific multidimensional pain scales such as the Glasgow composite measure pain scale (CMPS) for dogs [14] and cats [15] and the Colorado State University Feline and Canine Acute Pain Scale (CSU-FAPS; CSU-CAPS) (Colorado State University Cancer Centre, Veterinary Medical Centre 2006). To date a similar approach has not been used in rabbits.

Two recent surveys on the attitudes of veterinary surgeons [1] and veterinary nurses [16] towards pain and analgesia in rabbits highlighted that a large number of veterinary professionals did not use any pain assessment tool in rabbits (77% veterinary surgeons and 71.1% veterinary nurses). The majority of the respondents using a pain assessment tool stated they used the RbtGS regularly (12% and 20.5% of the overall population of veterinary surgeons and veterinary nurses respectively). None of the respondents mentioned the pain scale from Morton and Griffiths. The reason could be that, despite being a multidimensional pain scale, it is not specific to rabbits, and it is not currently used in other species such as cats and dogs as other composite pain scales are now available. The CANCRS was not mentioned as it was developed after the surveys were carried out. Other pain assessment tools were mentioned such as unidimensional pain scales, behavioural changes and multidimensional pain scales designed for other companion animals such as cats and dogs. However, all these methods either are not specific to rabbits, or they can be time consuming, and are less reliable than species-specific pain scales as they can be subjective to the observer’s experience [17, 18], leading to potential risks of undertreating rabbits in pain. Approximately half of veterinary nurses (50.35%) believed that a validated composite pain scale specifically designed for rabbits would improve pain assessment in this species [16].

The aim of this study was to develop a multidimensional pain scale specific for rabbits intended to assess acute pain in rabbit patients using a unique combination of methods: focus groups and behavioural observation. The purpose of the focus groups was to identify descriptors of a rabbit in pain using stakeholders in the field of rabbit medicine, and analgesia and anaesthesia following the methods described by Wong et al. [19] and Kinalski et al. [20]. Focus group discussion allows the determination of the best descriptors that can be used to construct a pain assessment tool that is specific to rabbits [21]. However, prey species like rabbits tend to hide signs of pain and therefore can be challenging for a practitioner to describe their behaviours properly [14]. Behavioural observation of the animals may be used to confirm pain descriptors and to identify additional behaviours that a rabbit in pain might exhibit [22] and this can be carried out by observation of video clips of rabbits recorded during the perioperative period [22]. Another important aspect when developing any scale, is to test its internal consistency, which can be assessed using the Cronbach’s alpha coefficient [23]. The coefficient is a measure ranging between 0 and 1, where results closer to 1 signify that there is good level of correlation between the categories measuring pain [24, 25]. It can also be used to assess whether the score given to each category by different respondents is consistent and free from random error [24].

Whilst previous rabbit pain scales have used facial action units and a combination of physiological and behavioural parameters extrapolated from cat and dog pain scales, a careful selection of the rabbit pain indicators based on focus group discussion and observation have not been previously carried out. Therefore, here we used a series of progressive steps to devise the new Bristol Rabbit Pain Scale.

## Methods and results

This study’s protocol and the written consent forms were approved by the Faculty of Health Sciences Research Ethics Committee (FREC-66205) of the University of Bristol.

**Fig 1:**
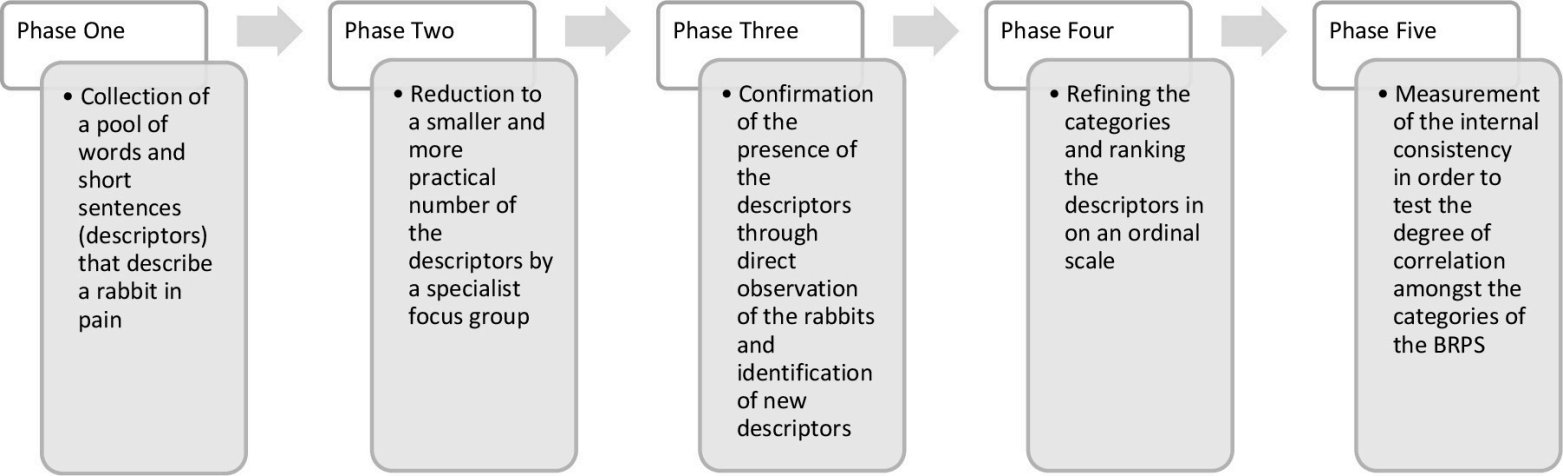
Overview of the BRPS development.

The overall development of the BRPS involved five phases as described below (Fig 1).

### Phase one: Collection of descriptors that describe a rabbit in pain

#### Methods

The aim of this phase was to identify words and short sentences (descriptors) that described rabbits in pain and to group them into categories. This was achieved with stakeholders in the fields of rabbit management and medicine.

A total of nine focus groups (total of 34 participants; 28 females and 6 males) were carried out during November 2018: One of veterinary nurses (6 people), two of rabbit owners (5 and 4 people respectively), two of rehoming centre workers (5 and 3 people respectively), one of scientists (3 people) and one of veterinary surgeons (8 people). The participants were contacted based on their knowledge and interest in rabbits and were recruited via email and were all based in the South-West of the United Kingdom (UK). They all gave written consent. The inclusion criteria for the veterinary surgeons, veterinary nurses, rehoming centre workers and rabbit owners were that they all had experience either treating, and nursing or looking after rabbits at the time the focus group was carried out. The inclusion criterion for the scientists was that they all had worked with rabbits during their scientific career.

Each focus group met once at the University of Bristol for two hours, during which a facilitator (LB) led the participants through three exercises. The aim of having different focus groups and exercises was to allow participants with the same background to discuss freely the topic with minimal intervention from the facilitator. The first exercise encouraged the participants to list a series of words and short phrases (descriptors) that described pain in rabbits. No limitation to the number of descriptors was applied. Descriptors were not prioritised or rejected during this phase. During the second exercise, descriptors from other research studies that used behaviour-based measures to assess pain in cats, rabbits and guinea pigs [15, 22, 26] were shown to the participants. The participants were then asked to select the descriptors that best applied to a rabbit in pain and add them to the previous list. In the third exercise, nine categories were provided (Posture, Comfort, Demeanour, Mobility, Response to touch, Facial expression, Attention to the painful area, Response to people, and Other). The category Facial expression was taken from the RbtGS [4] while the rest of the categories were taken from cat and dog pain scales [15, 27]. The participants were asked to group the descriptors collected during the first two exercises under these categories. The participants were encouraged to change the title of the categories or to add more categories if they wished to.

#### Results

When the descriptors were pooled together from the nine different focus groups, some descriptors were repeated in more than one category. A total of 276 descriptors and 12 categories were collected. None of the nine categories originally suggested were rejected. None of the groups changed the title of any of the categories including ‘Other’. However, three new categories, Response to other rabbits, Food and drinking, and Audible, were added (Table 1).

**Table 1:** Categories and descriptors collected during the nine focus groups in phase one of the study. Nine categories were provided (Posture, Comfort, Demeanour, Mobility, Response to touch, Facial expression, Attention to the painful area, Response to people, and Other) and three new categories were added by the participants (Response to other rabbits, Food and drinking, and Audible)*. Some descriptors were reported in more than one category.

Category	Descriptors
Comfort	Body adjustment, restless, bar chewing, teeth grinding, agitated, anorexia, distressed, drinking, eating, faecal pellets, groaning, growling, grunting, hiding, hunched, interacting with bottle, licking, look for comfort, lying on a side, not getting a comfortable position, not grooming self, overgrooming, prayer position, screaming, seeking softer surfaces, shaking, vocalisation
Posture	Hunched, tense posture, belly pressing, crouched, different posture, body adjustment, rigid, lay down legs under body, arching the back, ear position, flattened body, flattened ears, full body flex, lying on side, still, stretched out, stretching legs, cowering back of the cage, different position, fluffing up, fore legs extended, head down, laboured breathing, lay down hind legs extension, lay down legs to side, mouth breathing, muscle tensing, rearing, shaking, sitting, standing, stretching, tail lifting, tighten, tucked up, unusual position
Demeanour	Disinterested, indifferent, depressed, quiet, cowering back of the cage, dull, fearful, grumpy, agitated, anxious, unresponsive, withdrawn, biting, restless, aggressive, anorexia, frightened, lethargic, teeth grinding, asleep, bar chewing, change relationship with companion, defecating, digging, eating less, flinching, growling, hiding, hyper alert, immobile, inappropriate urination, less alert, less happy, less playful, licking, lunching, nervous, not alert, not grooming, not interacting with environment, reduced appetite, selective feeding, silent, thrashing, thumping, twitching, vocalisation, walking, wary, yawning
Mobility	Shuffling, staggering, still, reduced activity, walking, immobile, inactive, limping, not blinking, reluctant to move, avoiding using litter tray, avoiding using stairs/ramp, belly pressing, body adjustment, fore legs extended, frozen, hopping, hunched, jumping, lethargy, no caecotrophy, perineal soiling/scalding, restless, running, unresponsive
Response to touch	Flinching, vocalisation, flinching at touch, aggression, freezing, response to stroke, twitching, unresponsive, abdominal contraction, aggression when handled, biting, kicking out, rigid, shaking, squeaking, squealing, struggling, thumping
Facial expression	Closed eyes, ears flattened, squinting eyes, furrowed brows, third eyelid, sad, whiskers flattened, wide eyes, change in whiskers position, depressed, ears, eyes, facial grimace, glazed eyes, licking lips, nasal flare, pinched facial features, rapid nose movement, sweat around nose, teeth grinding, whiskers forward
Attention to the painful area	Licking wound/painful area, attention wound/painful area, biting wound/painful area, overgrooming, chewing, scratching, avoiding being touched, avoiding touching painful area, belly pressing, flinching, freezing, grinding teeth, guarding painful area, holding leg up, momentary lifting of rear paw, overgrooming sore area, scratching wound, struggling, unresponsive, vocalisation
Response to people	Aggressive, biting, hiding, avoidance, disinterested, isolate themselves, lashing out to a person, thumping, vocalisation, aggression towards owner, being needy towards people, change in interaction with the owner, cowering back of the cage, defecating, ears, eyes, flinching when handled, frightened, grumpy, grunting, less accepting handling, letting being handled, more accepting handling, passive, response to stroking, struggling, struggling when handled, unresponsive, wanting human attention
Response to other rabbits*	Aggressive, being needy, change in interaction with companion, not grooming companion, not wanting interaction,
Food and drinking*	Anorexia, eating less, lack of appetite, not drinking
Audible*	Screaming, teeth grinding, vocalising
Other	Anorexia, eating less, teeth grinding, shaking, changing toileting behaviour, abdominal contractions, asleep, avoiding contact, bar chewing, body twitching, bottle interaction, bullied, chewing, defecating, digging, disinterested, drinking more, fast breathing, giving up, grunting, hiding, lack of grooming, less exploratory, non-responsive to stimuli, not drinking, not interested in food, obvious trauma, reduced grooming, salivating, shaking head, trembling, urinating

### Phase two: Reduction of the descriptors and categories

#### Methods

The aim of this phase was to reduce the descriptors and categories collected during the previous phase to a smaller more practical number. This was carried out during one single specialist focus group attended by four participants (4 males): two specialist veterinary surgeons in rabbit and small mammal medicine (DZooMed) and two in small animal anaesthesia and analgesia (Dip.ECVAA). None of the participants of this focus group had attended the first focus group. All the participants gave a written consent. The participants were contacted based on their expertise in the field of rabbit medicine and small animal pain and analgesia. They were recruited via email and were all based in the South-West of the United Kingdom (UK). Due to their extensive knowledge, the specialists were considered the best judges to determine which categories and descriptors from the previous list should be used in the novel pain scale. However, descriptors and categories that were reported by the majority of the groups (≥ 6) during the previous phase were automatically included in the new list.

The specialists focus group met once at the University of Bristol in March 2019 for two hours and conducted three exercises facilitated by LB. During the first exercise, the specialists were provided with the list of categories from Phase 1 and asked, based on their clinical experience, to choose the categories that they thought should be included in the novel pain scale. Secondly, the specialists were provided with the list of descriptors from Phase 1 and were asked to select the descriptors for each of the previously named categories. During the third exercise, the specialists were asked to review the categories and descriptors that had so far been selected, and to add one or more descriptors describing ‘No-pain’ for each category. They were also requested to review the name of the categories.

#### Results

During the first exercise, the categories Demeanour and Posture were reported by the majority of the first focus groups and included in the new list. The categories Facial expression, Attention to the painful area, Audible and Other, were chosen by the specialists. During the second exercise, fifteen descriptors were reported by the majority of the first focus groups and included in the new list. Fifty-seven descriptors were selected by the specialists and added to the new list. The total of 72 descriptors were then grouped under the six categories. During the third exercise, the category ‘Other’ was renamed ‘Food intake/toileting’. The category Posture was renamed Posture/Locomotion. A total of 10 ‘No pain’ descriptors were added (Table 2).

**Table 2:** Final descriptors and categories chosen during the specialist focus group in phase two. The ‘No Pain’ descriptors added during the focus group are presented in *Italic*.

**Demeanour**
*Alert/responsive*
Depressed, Quiet, Hiding, Cowering at the back of the cage, Disinterested, Unresponsive, Biting, Aggressive, Dull, Grumpy, Restless, Lethargic, Withdrawn, Allow to be handled, Lashing out to a person, Lunging, Stop grooming themselves, Isolate
**Posture/Locomotion**
*Relaxed/comfortable*
Hunched, Tense posture, Belly pressing, Reluctant to move, Still, Inactive, Reduced mobility
**Facial Expression**
*Open eyes*
Flattened ears, Squinting eyes, Furrowed brows, Flattened whiskers, Wide eyes, Eyes tightened, Frowning, Nasal flare, Whiskers forward/stand-out
**Food intake/toileting**
*Eating (No pain)*, *Passing urine/faeces (in the litter tray)*
Anorexia, Lack of appetite, Eating less, Reduced appetite, Altered toileting, Inappropriate urination, Perineal soaking, Reduced faeces, No faeces
**Attention to the painful area**
*Lack of attention/ignoring*
Licking the wound/painful area, Biting the wound/painful area, Chewing the wound/painful area, Attention to the wound/painful area, Overgrooming, Flinching as response to palpation, Reaction as response to palpation
**Audible**
*Silent*
Screaming, Teeth grinding, Vocalising, Grunting, Groaning, Growling, Squealing

### Phase three: Video observation

#### Methods

The aim of this phase was to confirm through direct observation of rabbits undergoing surgery that the descriptors identified in Phases 1 and 2 as denoting pain, did occur more post-surgery, and those denoting no pain were more likely to occur pre-operatively and, as a consequence, to reject those terms that were ambiguous. A second aim was to identify any additional behaviours exhibited by rabbits undergoing surgery that could be used to construct the novel pain scale but that had not been included in the previous list of descriptors.

Thirty-one rabbits of any age, gender and breed undergoing ovariohysterectomy (OVH) and orchiectomy (OR) from four veterinary clinics in the South-West of the United Kingdom (UK) were video and audio-recorded using GoPro Hero7 Black® Cameras during the perioperative period from the moment they were hospitalised until the time they were discharged on the same day. The cameras were securely fitted on the door of the kennel, facing the rabbits with a maximum distance of 700 mm to the back of the kennel. The rabbits were hospitalised in the wards either alone or with other animals, and veterinary staff would enter the room intermittently. The owners had given consent to inclusion in the study and analgesia was provided to the rabbits according to the normal practice protocol.

The videos from three rabbits were rejected (one was considered too poor quality and two showed two rabbits housed together), so 28 individually-housed animals were included in this study. Four video clips of 5-minute-length each were selected per rabbit; two before (At 30- and 60-minutes post admission) and two from after surgery (At 120- and 150-minutes of the recovery time after tracheal extubation). A total of 112 video clips were analysed (n = 56 Before surgery-B; n = 56 After surgery-A). The video clips were randomised, and the observer (LB) was blinded as to whether each video clip was ‘B’ or ‘A’. The presence of each descriptor listed during Phase 2, including the no-pain ones (Table 2), was assessed in each video clip. If the descriptor was observed, it was recorded as one (1) if it was not observed it was recorded as zero (0). If any additional behavioural descriptors were observed in the video clips, they were added to the list, and the presence or absence recorded in each video clip.

#### Results

In this phase, 27 descriptors were rejected because their definition was ambiguous and difficult to interpret e.g. grumpy, furrowed brows (n = 11), they described changes over time and therefore could not be quantified during a short assessment period e.g. eating less, reduced faeces (n = 6), or they implied a relationship or action towards another rabbit or a person and therefore could not be assessed during the observation as the animals were hospitalised individually, e.g. lashing out to a person, flinching as response to palpation (n = 10).

None of the descriptors in the categories Attention to the painful area (n = 5) and Audible (n = 7) were recorded in any of the video clips and therefore these categories were rejected. The descriptors that occurred both before and after surgery and did not show any difference between the two periods were also rejected (n = 7; Table 3).

**Table 3:** Results from the analysis of the video clips. The number in each column (Before; After) corresponds to the number of video clips in which each descriptor was observed. Some of the descriptors were rejected because ^a^ they were ambiguous (n = 11); ^b^ they described changes over time (n = 6); ^c^ they implied relationships with or action towards another rabbit or a person (n = 10); ^d^ they were not seen in any of the video clips (n = 12); ^e^ they did not differentiate between before and after surgery (n = 7). The new descriptors were recorded under the categories “New descriptors”.

	BEFORE	AFTER
**Demeanour **		
*Alert/responsive (No pain word) *	50	2
Depressed	0	7
Quiet	5	43
Disinterested	0	5
Dull	1	8
Lethargic	0	8
NOT grooming themselves	30	47
Hiding ^e^	5	4
Rejected: Unresponsive ^d^ Cowering at the back of the cage^a^, Biting^c^, Aggressive^c^ Grumpy^a^ Restless^a^, Lashing out to a person^c^, Lunging^c^, Isolate^c^, Withdrawn^a^, Allow to be handled ^c^		
**Posture/Locomotion **		
*Relaxed/comfortable (No pain word) *	47	0
Hunched	1	18
Tense posture	1	12
Still	7	47
Inactive	7	44
Rejected: Belly pressing ^d^ Reluctant to move^c^, Reduced mobility ^b^		
**Facial Expression **		
*Open eyes (No pain word) *	49	12
Flattened ears	0	3
Eyes tightened	0	3
Rejected: Whiskers forward/stand-out ^e^ Squinting eyes^a^, Furrowed brows^a^, Flattened whiskers^a^, Wide eyes^a^, Frowning^a^, Nasal flare^a^		
**Food intake/toileting **		
*Eating (No word pain) *	23	0
*Passing urine/faeces (in the litter tray) (No pain word) *	12	7
Anorexia	29	50
Rejected: No faeces ^e^ Lack of appetite, Eating less ^b^, Reduced appetite ^b^, Altered toileting ^b^, Inappropriate urination ^b^, Perineal soaking^c^, Reduced faeces ^b^		
Attention to the painful area*		
Rejected: *Lack of attention/ignoring (No pain word)* ^e^, Licking the wound/painful area ^d ^, Biting the wound/painful area ^d^, Chewing the wound/painful area ^d ^, Attention to the wound/painful area ^d^, Overgrooming ^d^, Reaction as response to palpation^c^, Flinching as response to palpation^c^		
Audible*		
Rejected: *Silent (No pain word)* ^e,^ Screaming ^d^, Teeth grinding ^d^ , Vocalising ^d^ , Grunting ^d^, Groaning ^d^, Growling ^d^ , Squealing ^d^		
**New descriptors**		
Grooming	20	0
Moving ears	42	2
Not moving ears	8	46
Exploring /active	28	1
Moving a little	18	8
Semi-closed eyes	4	25
Closed eyes	0	13

The rest of the descriptors were confirmed as potential descriptors for the novel pain scale. The no-pain descriptors of four categories (Demeanour, Posture/Locomotion, Facial expression and Food intake/toileting) were recorded predominantly before surgery. The rest of the descriptors were recorded predominantly in the after-surgery video clips. Additional descriptors were identified and grouped under a category called “New descriptors”: semi-closed and closed eyes, moving and non-moving ears, exploring/being active, mobility, laying on a flank, and grooming (Table 3).

### Phase four: Ranking descriptors of the BRPS

#### Methods

The aim of Phase 4 was to refine the categories and rank the descriptors in order of pain intensity using a 4-point scale. The categories from the previous phase, were reviewed by the authors and additional changes made in order to better define them and increase clarity. The new descriptors identified from the video observation were also allocated within the refined categories. Finally, the descriptors within each category were ranked in an ordinal scale with four levels of pain intensity. To ensure correct ranking, descriptors from previous studies on pain in rabbits were used as a guideline [22, 28, 29] by the authors (LB, JM, NJR). Video observation of the rabbits was also used to adjust the descriptors for accuracy and to transform them into short sentences to better describe the differences that a rabbit would exhibit depending on the intensity of pain.

#### Results

The category Posture/locomotion comprised five descriptors: three describing posture [Relaxed/Comfortable, Hunched, Tense posture) and two locomotion (Still, Inactive), and was split into two new categories: Posture and Locomotion. The category Facial expression contained three descriptors (Open eyes, Flattened ears and Eyes tightened) and was changed into two new categories “Ears” (Flattened ears) and “Eyes” (Open eyes, Eyes tightened). The category Food intake/toileting was changed into “Food” as the descriptors describing the faecal output had been previously rejected. A new category “Grooming” was added. The new descriptors identified from the video observation were allocated within the new categories: Semi-closed and Closed eyes (Eyes); Moving and Not moving ears (Ears), Exploring/being active, Mobility (Locomotion), Laying on a flank (Posture) and Grooming (Grooming).

The descriptors within each of the final seven categories (Demeanour, Locomotion, Posture, Ears, Eyes, Food and Grooming) were then ranked on an ordinal scale. The no-pain descriptors were listed under the intensity pain “0” (No Pain). The remaining descriptors were ranked from 1 to 3. The categories Posture, Ears, Grooming and Food listed fewer descriptors than the four levels of pain intensity so the gaps were filled with new descriptors (Table 4).

**Table 4:** Ranking of descriptors on an ordinal scale. The descriptors in bold were added in order to fill the gaps due to a small number of descriptors within the category.

	0 (No pain)	1 (Mild pain)	2 (Moderate Pain)	3 (Severe pain)
**Demeanour**	Alert/responsive	Disinterested, Quiet	Dull, Lethargic	Depressed
**Locomotion**	Exploring/Active	Moving a little	Inactive	Still
**Posture**	Relaxed/comfortable	**Sitting or lying**	Hunched	Tense Posture
**Ears**	Moving ears	**Slightly moving ears**	Not moving ears	Flattened ears
**Eyes**	Open eyes	Semi-closed eyes	Closed eyes	Eyes tightened
**Grooming**	Grooming	**Grooming little**	**Grooming with little energy**	Not grooming
**Food**	Eating	**Interest in food but limited foraging**	**Interest in food but not foraging**	Anorexia

### Phase five: Internal consistency

#### Methods

The aim of this phase was to measure the internal consistency of the BRPS using the Cronbach alpha coefficient, which describes the extent to which all the categories measure the same concept [25]. A total of 21 respondents, veterinary surgeons and veterinary nurses working with rabbits, were contacted by email and provided with the novel BRSP in the format of a one single page form and were asked to assess up to four rabbits in acute pain using the scale. They were also asked to give general comments regarding the use of the novel scale. The internal consistency of the BRPS was calculated using the Cronbach alpha coefficient and analysed using SPSS (Version 20, IBM). The coefficient is generally measured between 0 and 1 and a coefficient ≥ 0.7 is considered good internal correlation [24, 25]. The impact of removing each category from the scale was also quantified.

#### Results

A total of 61 rabbits in acute pain were assessed. The rabbits had been admitted for a variety of conditions such as mild trauma, intestinal hypomotility and surgical procedures such as OVH and OR. All four levels of pain were represented with a median score (and range) of 8 [0–18]. Cronbach’s alpha coefficient of the BRSP was 0.846. The removal of each category had similar impact on the internal consistency (Table 5). In view of the comments expressed by some of the respondents regarding food being a limitation within a clinical setting, the category Food was removed from the final version of the scale. This change did not affect the overall internal consistency of the BRPS (alpha = 0.843) (Table 5). The BRPS was then finalised and instructions on how to use it added to the ultimate version (Table 6).

**Table 5:** The internal consistency of the BRPS was analysed using the Cronbach’s alpha test. The table shows the Cronbach’s alpha of the scale if each item would be deleted.

Category	Cronbach’s alpha if item deleted
Demeanour	0.814
Posture	0.804
Locomotion	0.795
Ears	0.824
Eyes	0.852
Food	0.843
Grooming	0.834

**Table 6:** Final version of the Bristol Rabbit Pain Scale (BRPS).

Categories	0	1	2	3
Demeanour	The rabbit is looking around, is alert and responsive to the surrounding environment OR the rabbit is asleep	The rabbit is awake but shows little interest in the surrounding environment	The rabbit is dull and is not responsive to the observer or surrounding environment	The rabbit is unresponsive to the observer or surrounding environment even if approached
Locomotion	The rabbit is active and hopping around the area. OR is relaxed or asleep	The rabbit appears hesitant to move and shows little activity	The rabbit is inactive and does not move during the observation period, except when approached	The rabbit is inactive and does not move at all, even when approached
Posture	The rabbit is resting in a relaxed and comfortable posture e.g. lying on a flank or on its front with its hind legs to the side, or is moving freely. OR the rabbit is asleep	The rabbit is sitting or lying on its front with visible fore-legs	The rabbit is sitting or lying on its front with its legs under its body and appears hunched	The rabbit is sitting or lying on its front with its legs under its body, and the body looks tense, stiff and hunched OR the rabbit is pressing its abdomen against the ground
Ears*	The rabbit moves its ears freely and turns them towards sounds. OR the rabbit is asleep	The rabbit moves and slightly turns its ears towards sounds	The rabbit does not obviously move its ears, but reacts slightly to sounds (e.g. with a head turn)	The rabbit does not move its ears at all and does not react to sounds OR the ears are flattened against its back
*Take in consideration that lop-eared rabbits may show less pronounced changes				
Eyes	The rabbit has its eyes open. OR the rabbit is asleep	The rabbit keeps its eyes semi-closed	The rabbit keeps its eyes closed	The rabbit keeps its eyes closed and tightened
Grooming	The rabbit is meticulously grooming his/herself. OR the rabbit is asleep	The rabbit is grooming but gets distracted easily	The rabbit is attempting grooming, but with little energy	The rabbit is not grooming at all

How to use the BRSP

1) Observe the rabbit for 3 minutes.

2) Then quietly approach the cage before scoring the behaviours described in each category.

3) Score each category on a 0–3 scale based on the behaviours that the rabbit exhibits for most of the observation.

4) Calculate the total score from 0–21.

## Discussion

The current study describes the development of the Bristol Rabbit Pain Scale (BRPS), a multidimensional pain scale specific to rabbits. The development process consisted of a total of five phases, using a unique combination of methods: focus groups and behavioural observation. During the first two phases, focus groups were used to obtain a pool of descriptors of rabbits in pain that could be added to the novel pain scale. The third phase consisted of video observations of rabbits during the perioperative period to confirm the descriptors included in the novel pain scale and to reject those that were ambiguous. The last two phases focused on the construction of the final format of the novel pain scale, formatting it, and testing its internal consistency. This led to a composite pain scale of six categories (Demeanour, Posture, Locomotion, Ears, Eyes and Grooming) and four intensities of pain (0, 1, 2, and 3) with a total score from 0 to 18.

Similar, to the RbtGS [4] and the CANCRS [9], the BRPS is a pain assessment tool specific for rabbits, and like the CANCRS is a composite pain scale. The RbtGS followed the method used to develop the rat [30] and mouse [31] grimace scales, which obtained still images of the head and face of rabbits before and after painful procedures such as ear tattooing, and to score the facial expressions on a 3-point- scale. The CANCRS was developed by merging the RbtGS with physiological and behavioural responses (CPS) selected from pain scales used to assess cats and dogs [9]. In contrast, the BRPS was developed following two distinct methods, the method used to develop the Glasgow composite measure pain scale (CMPS) for cats and dogs [15, 27, 32] that comprises collection and expert validation of the descriptors [33], and video observation of the animals to confirm the descriptors more commonly exhibited by animals in pain during the perioperative period.

### Focus groups

The descriptor collection and expert validation were carried out by focus groups. The use of focus group discussion is a well-recognised method described in both human and veterinary medicine to gather information about a specific topic [19, 34, 35]. In human medicine, it is often incorporated into the development of pain scales for subjects that are unable to self-report pain such as children [36, 37] and individuals with difficulties in verbal communication [38]. This is generally carried out during several sessions when the indicators are carefully discussed amongst the content experts such as clinicians and nurses specialised in the specific area. Studies have shown that observers are also capable of describing behavioural changes seen in animals due to pain [33]. In veterinary medicine, this method was first used by Morton and Griffiths in 1985 [13] and then consolidated during the develop of the CMPS for dogs [32] and cats [15]. Here, uniquely, we used focus groups with experts from a variety of disciplines, owners and researchers as well as veterinary professionals which we believed served to ensure diverse potential indicators of pain behaviours were all considered.

### Video observation

In the current study, video observation of rabbits was used to further assess the descriptors generated during the focus groups; to confirm viability of those included in the novel pain scale and to reject those that were ambiguous. Similarly, in human medicine, novel pain scales developed to assess non-verbal patients are often tested through direct observation of the patient in order to find the descriptors that best describe the response of the subject to pain [37, 39, 40]. In veterinary medicine, direct observation using video recording is generally reported in studies to assess post-operative pain in animals such as rats [41], guinea pigs [26], rabbits [42]and horses [43]. In these studies, ethograms, created using information from the literature review, were used to assess the most common pain behaviours exhibited by the animals.

The video analysis of the rabbits led, amongst other changes, to the rejection of all the descriptors in the category ‘Audible’ that described an animal vocalising, and the category ‘Attention to the painful area”. This was because none of these descriptors were recorded in any of the video clips suggesting that their inclusion added no value.

In a study on the use of local anaesthetic cream for pain-free venepuncture in laboratory animals [44], vocalisation was used to assess pain in dogs and rats, but not in rabbits. In contrast, Morton and Griffiths (1985) [13] described vocalisation as a sign of pain, distress or discomfort not only in laboratory animals such as dogs, cats, guinea pigs and rats but also in rabbits. Similarly, vocalisation was observed in rabbits experiencing pain in a study evaluating EMLA® cream use during tattooing of rabbits [4], and when tested for inter-rater reliability, the results showed a good level of agreement (k = 0.88) [9]. In the current study, none of the rabbits vocalised during the perioperative period. One explanation could be, that rabbits tend to vocalise when short and sharp acute pain is inflicted such as ear tattooing, and they do not vocalise when suffering acute post-operative pain. It is possible that more research needs to be done to better describe vocalisations when assessing pain in rabbits, and to determine the use of this category in pain scales depending on their specific purpose.

The category ‘Attention to the painful area’ is frequently used to assess pain in cats [15], dogs [14] and horses [45]. In these species, if the animals are pain-free they tend not to pay attention to a wound while if they experience severe pain, depending on the species, they can chew the wound, growl or become aggressive when the wound is palpated. Rabbits respond differently. In a study that evaluated EMLA cream attention to the painful area was identified by the rabbit grooming the head and ears following ear tattooing [4]. During the development of the CANCRS, neither the category ‘Attention to the painful area’ nor ‘Grooming’ were used as indicators of pain but ‘Response to palpation’ was included as a category. This was chosen based on other pain scales for small mammals and rated from 0 to 2 where 0 was no reaction, 1 was reaction during palpation and 2 was reaction before palpation [9]. In the current study, both categories ‘Attention to the painful area’ and ‘Response to touch’ were initially highlighted by the first focus group but ‘Attention to the painful area’ was then favoured over ‘Response to touch’ by the specialist focus group. However, in the video analysis, none of the rabbits paid attention to the surgical wound and therefore the category ‘Attention to the painful area’ was rejected.

Grooming was seen to be a more common response of the animal to pain: the grooming of the head and body was observed more frequently in rabbits with no or mild pain, but it was not exhibited by the animals suffering more severe pain. These findings were confirmed by a recent study on post-operative behaviour in 28 rabbits undergoing orthopaedic surgery where the duration of body self-cleaning was found to be shorter during pain (1 hour post recovery) than at 24 h post recovery [42]. This study also assessed the influence of the presence of an observer on pain behaviours and found that no significant difference was found in body cleaning while ear scratching and head self-cleaning did not change between the presence and absence of an observers. This is an important consideration as the presence of veterinary staff assessing the animals could have altered this behaviour. Grooming is a normal behaviour exhibited by rabbits as part of their daily routine [46, 47] but non-essential behaviours such as this normally cease in cases of stress or danger [48]. In addition, previous studies suggest that overgrooming of the painful area can be seen following sharp and acute pain [4]. Therefore, ‘Grooming’ was added as category.

The categories Demeanour, Posture and Locomotion were included in the final version of the BRPS. These categories were previously reported in studies assessing pain in rabbits [28], cats [15] and dogs [32]. An animal in pain usually tends to be less responsive and to exhibit a tense posture. However, while predator species such as cats and dogs are described as restless or reluctant to move depending of the intensity of the pain [15, 32], prey species such as guinea pigs and rabbits are generally less active and less mobile when in pain [4, 26]. The CANCRS includes the response of the animal to the environment and external stimuli under the category Mental status, it does not include Posture or Locomotion [9]. The BRSP reflects these species differences in pain response and takes into consideration changes that are specific to rabbits.

The BRPS also includes the categories ‘Ears’ and ‘Eyes’.; two facial action units previously reported in the RbtGS, in other grimace scales such as those used on rats and mice [30, 31] and in behaviour-based studies on the perioperative pain in rabbits [28]. In the CANCRS, these two facial units were found to have substantial inter-rater agreement (Cohen’s kappa: ear position = 0.78, and orbital tightening = 0.68). When describing orbital tightening, the current study confirmed findings of the previous studies, that an animal suffering some degree of discomfort and pain keeps their eyes semi-closed or closed whilst ones pain-free keeps their eyes open.

Generally, when assessing the ears to measure pain, the position is taken into consideration and a rabbit in pain holds the ears flat against the body [4]. During the current study, the ear position was considered alongside ear movement as, like other animals rabbits tend to move their ears towards a sound or a noise [49]. During the preoperative period, rabbits were more likely to orient their ears in response to a noise, while after surgery, they kept the ears still. Similarly, responsiveness to sounds was taken in consideration when developing a pain scale for acute colic in horses, where the ear orientation towards sounds was considered 0 (no pain) and no response to sounds and backward position was scored 2 (intense pain) [50]. One advantage of evaluating ear position and movement together, is that it also allows assessment of lop-eared rabbits. Lop-eared, like straight-eared rabbits, tend to orientate the ears towards sounds although to a smaller degree. During the direct observations, it was noticed that mini-lop and dwarf lop rabbits moved the lopped pinna backwards and forwards, while rabbits with heavier ears such as French lop rabbits exhibited subtle, but obvious movements of the base of the ears and of the head but were unable to move the pinna. The BRPS takes into consideration changes including these two facial action units.

### Internal consistency

The Cronbach’s alpha coefficient of the BRPS was good at 0.843. This is similar to the multidimensional pain scale developed to assess acute postoperative pain in cats (alpha = 0.867) [51] and higher than the Glasgow scale for assessing pain in dogs (alpha = 0.632) [32]. Neither the RbtGS nor the CANCRS were tested for internal consistency. The Cronbach’s alpha coefficient of the BRPS showed that all the categories had good level of correlation between them. The test also showed that none of the categories, if deleted, would have substantially changed the alpha coefficient and therefore the internal reliability of the scale.

### Limitations

One limitation of the current study is that the rabbits observed during the direct analysis of Phase 3 underwent either ovariohysterectomy or orchiectomy, two surgical procedures considered mildly to moderately painful [1]. Therefore, it is possible that during the direct observations, changes associated with more severe pain were not seen in the videos. However, although the use of analgesia and multimodal analgesia has increased over the years in rabbits [1], many analgesic protocols have not been tested in this species and many studies have shown that an optimal analgesic protocol has not yet been found [5, 52, 53]. Based on this, it is reasonable to assume that the rabbits in the current study might have experienced some post-operative pain, and some severe pain.

Moreover, it is reasonable to assume that due to the administration of analgesia, during the perioperative period following the normal protocol of the veterinary practice, the rabbits may have not exhibited some pain behaviours limiting the pain descriptors analysed and used in the development of this novel pain scale.

To be able to adequately describe the different level of pain during the ranking of the descriptors, the choice of the descriptors for each category was guided by the experience of the authors (LB, JM and NJR), similarly to other studies [51]. The choice of the descriptors was also guided by a literature review of studies describing pain in rabbits [22, 28, 29] and confirmed by comparing the video clips before and after surgery. This method was previously reported during the development of a tool to measure pain in children in an emergency department [39] in order to select words and descriptors for inclusion in their pain scale for children, analysed and compared scales previously validated for the youngest patients. Current paucity of multidimensional pain scales in rabbits meant this was not possible and therefore we relied entirely on reviewing the literature [21].

Another limitation of the current study is that the rabbits that were video recorded during the peri-operative period had interaction with veterinary staff. This could have contributed to some changes of the pain behaviours. Pinho et al. (2020) assessed pain-related behaviours in rabbits following orthopaedic surgery and the effects of the presence of an observer. They found that, during the pre-operative period, the rabbits were less active and explored less if the observer was present while during the post-operative period, behaviours such as e.g. winching increased and flinching decreased. They concluded that the influence of an observer on the rabbit’s behaviour could lead to false negative results when assessing pain. In a busy veterinary clinic, the interaction between the animals and the veterinary staff during the peri-operative period is normal practice. Veterinary staff is constantly responsible for the care of the inpatients while hospitalised. The aim of the current study was to develop a pain assessment tool that could be used in such environment. For this reason, the rabbits recorded in the videos were hospitalised in wards according to the normal practice protocol. Moreover, the video clips selected during the current study were solely time-based (Before and After surgery) to highlight those behaviours that were seen more frequently perioperatively irrespective of whether there was interaction or not between the rabbits and the veterinary staff.

The rabbits recorded in the videos during the pre-operative period could also have exhibited signs of stress due to the new environment leading to a misinterpretation of the “no-pain” descriptors. To reduce this eventuality, the “no pain” descriptors assessed during the video observation were based on the results of the two focus groups where specific pain behaviours were thoroughly considered. However, at the present, no stress scales such as those for horses [54] and cats [55] are available for rabbits, and therefore specific stress descriptors could not be confirmed or rejected during the analysis. The influence of stress on pain and the consequent rabbit behavioural response is not yet well understood, and further research is necessary.

## Conclusions

Adequate pain assessment is best achieved by the use of a species-specific, multidimensional pain scale. This study has filled a gap in rabbit medicine by developing the BRPS: a multidimensional pain scale similar to those currently available for cats and dogs, but specific to rabbits. The BRPS was developed using a unique approach based on focus group discussions and direct observation of the animals. It is comprised of six categories each rated on a 4-point scale and shows high internal consistency. The BRPS includes the category ‘Ears’ acknowledging that not only the position, but also the movement of the rabbit’s ears are important, and allowing the assessment of lop-eared as well as straight-eared rabbits, a limiting factor in other pain assessment tools already available for this species. The BRPS has the potential to improve the assessment and management of pain in rabbits by providing veterinary professionals with a novel, multidimensional pain assessment tool. Further studies will investigate the clinical utility, validity and reliability of the BRPS.
