# Correction: Development of the Bristol Rabbit Pain Scale (BRPS): A multidimensional composite pain scale specific to rabbits (*Oryctolagus cuniculus*)

**DOI:** 10.1371/journal.pone.0308219

**Published:** 2024-07-29

**Authors:** Livia Benato, Joanna Murrell, Toby G. Knowles, Nicola J. Rooney

There is an error in the caption for Table 6. The correct caption under “How to use the BRSP” is: Calculate the total score from 0–18. Please see the correct Table 6 caption here.

**Table 6:** Final version of the Bristol Rabbit Pain Scale (BRPS).

Categories	0	1	2	3
Demeanour	The rabbit is looking around, is alert and responsive to the surrounding environment OR the rabbit is asleep	The rabbit is awake but shows little interest in the surrounding environment	The rabbit is dull and is not responsive to the observer or surrounding environment	The rabbit is unresponsive to the observer or surrounding environment even if approached
Locomotion	The rabbit is active and hopping around the area. OR is relaxed or asleep	The rabbit appears hesitant to move and shows little activity	The rabbit is inactive and does not move during the observation period, except when approached	The rabbit is inactive and does not move at all, even when approached
Posture	The rabbit is resting in a relaxed and comfortable posture e.g. lying on a flank or on its front with its hind legs to the side, or is moving freely. OR the rabbit is asleep	The rabbit is sitting or lying on its front with visible fore-legs	The rabbit is sitting or lying on its front with its legs under its body and appears hunched	The rabbit is sitting or lying on its front with its legs under its body, and the body looks tense, stiff and hunched OR the rabbit is pressing its abdomen against the ground
Ears* *Take in consideration that lop-eared rabbits may show less pronounced changes	The rabbit moves its ears freely and turns them towards sounds. OR the rabbit is asleep	The rabbit moves and slightly turns its ears towards sounds	The rabbit does not obviously move its ears, but reacts slightly to sounds (e.g. with a head turn)	The rabbit does not move its ears at all and does not react to sounds OR the ears are flattened against its back
Eyes	The rabbit has its eyes open. OR the rabbit is asleep	The rabbit keeps its eyes semi-closed	The rabbit keeps its eyes closed	The rabbit keeps its eyes closed and tightened
Grooming	The rabbit is meticulously grooming his/herself. OR the rabbit is asleep	The rabbit is grooming but gets distracted easily	The rabbit is attempting grooming, but with little energy	The rabbit is not grooming at all

How to use the BRSP

1) Observe the rabbit for 3 minutes

2) Then quietly approach the cage before scoring the behaviours described in each category

3) Score each category on a 0–3 scale based on the behaviours that the rabbit exhibits for most of the observation

4) Calculate the total score from 0–18
